# Aptamers for Addressed Boron Delivery in BNCT: Effect of Boron Cluster Attachment Site on Functional Activity

**DOI:** 10.3390/ijms24010306

**Published:** 2022-12-24

**Authors:** Darya S. Novopashina, Maya A. Dymova, Anna S. Davydova, Mariya I. Meschaninova, Daria O. Malysheva, Elena V. Kuligina, Vladimir A. Richter, Iaroslav A. Kolesnikov, Sergey Yu. Taskaev, Mariya A. Vorobyeva

**Affiliations:** 1 Institute of Chemical Biology and Fundamental Medicine, Siberian Division of Russian Academy of Sciences, 630090 Novosibirsk, Russia; 2 Budker Institute of Nuclear Physics, Siberian Division of the Russian Academy of Sciences, 630090 Novosibirsk, Russia; 3 Department of Physics, Novosibirsk State University, 630090 Novosibirsk, Russia

**Keywords:** cell-specific aptamers, boron clusters, boron neutron capture therapy, boron delivery agents, glioblastoma, cancer treatment

## Abstract

Among the great variety of anti-cancer therapeutic strategies, boron neutron capture therapy (BNCT) represents a unique approach that doubles the targeting accuracy due to the precise positioning of a neutron beam and the addressed delivery of boron compounds. We have recently demonstrated the principal possibility of using a cell-specific 2′-F-RNA aptamer for the targeted delivery of boron clusters for BNCT. In the present study, we evaluated the amount of boron-loaded aptamer inside the cell via two independent methods: quantitative real-time polymerase chain reaction and inductive coupled plasma–atomic emission spectrometry. Both assays showed that the internalized boron level inside the cell exceeds 1 × 10^9^ atoms/cell. We have synthesized *closo*-dodecaborate conjugates of 2′-F-RNA aptamers GL44 and Waz, with boron clusters attached either at the 3′- or at the 5′-end. The influence of cluster localization was evaluated in BNCT experiments on U-87 MG human glioblastoma cells and normal fibroblasts and subsequent analyses of cell viability via real-time cell monitoring and clonogenic assay. Both conjugates of GL44 aptamer provided a specific decrease in cell viability, while only the 3′-conjugate of the Waz aptamer showed the same effect. Thus, an individual adjustment of boron cluster localization is required for each aptamer. The efficacy of boron-loaded 2′-F-RNA conjugates was comparable to that of ^10^B-boronophenylalanine, so this type of boron delivery agent has good potential for BNCT due to such benefits as precise targeting, low toxicity and the possibility to use boron clusters made of natural, unenriched boron.

## 1. Introduction

Despite tremendous progress in the development of new approaches to the treatment of malignant diseases, there are still a number of tumors that cannot be cured by any therapeutic means available in clinics at the moment.

Boron neutron capture therapy (BNCT) represents a unique combination of radio- and chemotherapy. It is based on the irradiation of boron-10 saturated tumor cells by an epithermal neutron beam that leads to selective cell killing [1]. BNCT doubles the accuracy of cell targeting due to: (1) the precise positioning of the neutron beam and (2) the addressed delivery of boron compounds into tumor cells. Importantly, BNCT is suitable for the treatment of tumors that are resistant to any other therapeutic options. In particular, glioblastoma is an aggressive malignant brain tumor, characterized by rapid and invasive growth that remains almost incurable. To date, there have been a number of examples of the successful use of BNCT for the treatment of brain tumors in clinics [2,3,4].

Clinical BNCT application depends on two key issues: (1) the generation of a neutron beam with suitable parameters and the possibility of precise focusing and (2) the availability of ^10^B-containing compounds specifically targeted to the tumor cells. The problem of attaining a neutron beam with the desired characteristics has been solved by several research teams. In particular, the Budker Institute of Nuclear Physics (BINP) (Novosibirsk, Russia) developed an accelerator-based epithermal neutron source VITA (Vacuum Insulation Tandem Accelerator) that is highly suitable for BNCT [5]. The search for targeted boron-10 compounds is now extensively ongoing. A broad variety of boron-loaded addressed biomolecules and complex delivery vehicles such as dendrimers, liposomes, nanotubes and nanoparticles (see [6,7] for recent in-depth reviews) has been developed up to now and has shown encouraging results in preliminary studies. However, none of them has yet been approved for clinical applications. The only boron-containing drugs now used for BNCT in clinics are based on boronophenylalanine (such as Steboronine^®^ from Stella Pharma [8]) or sodium borocaptate [2]. These compounds cannot be precisely addressed to the tumor, and their pharmacokinetics and biodistribution are not optimal, so their tumor uptake is characterized by significant variability [9].

Cell-specific oligonucleotide aptamers appear to be very promising delivery vehicles for BNCT. They can be targeted to the certain types of tumor cells [10], and the chemical nature of nucleic acids allows for plenty of possibilities to load aptamers with boron compounds. We have shown previously that the covalent attachment of a boron cluster to the 5′-end of a 2′-F-RNA aptamer specific to U-87 MG human glioblastoma cells generates an agent capable of inhibiting cell growth after irradiation by epithermal neutrons [11]. Further developing this research, we set ourselves the task of revealing which particular site of boron cluster attachment better preserves functional activity of the aptamer and finding out if there are any common rules for different aptamers. Moreover, in this study, we also evaluated the amount of boron atoms brought into the cell via aptamer-addressed delivery.

In our studies, we employed the tumorigenic human glioblastoma cell line U-87 MG as target cells. To compare the effects of model BNCT on cancerous cells versus non-cancerous cells, we also needed another cell line as a negative control. Undoubtedly, an ideal cell model as a negative control for studying the effect of targeted drugs on tumors is the normal tissue of the organ from which this tumor actually originates. Such primary cultures are desirable since they most precisely replicate the properties of normal cells in vivo. However, unlike immortalized cell lines, obtaining and culturing human neuronal primary cells is a very difficult task. Primary cell cultures are not immortal, which imposes additional experimental limitations [12]. Due to these difficulties in obtaining a primary cell culture of healthy brain tissue, we decided to use normal human fibroblasts. They are often used as a negative control in studies on oncology [13,14,15], stemness [16] and other nosologies [17]. Taking into account that fibroblasts are of mesenchymal origin, and glioma is of neuroectodermal origin, we considered also the use of pheochromocytoma rat cell line PC 12, an established model system for neuronal neurosecretion and differentiation. In particular, it is used to study cellular responses to nerve growth factors (NGFs) and how these lead to the expression of specific proteins and differentiation [18,19]. Otherwise, PC 12 cells are not of human origin. Moreover, NGF-induced neurite outgrowth from PC 12 cells involves the activation of the receptor tyrosine kinase TrkA PI3K/Akt signaling pathways [18], which are also activated in glioma cells. Taking all these factors into account, we finally chose human fibroblasts hFF8 as the control non-cancerous cells.

We have shown that the localization of a boron cluster must be chosen individually for each aptamer. It was found that targeted delivery driven by an aptamer provides cell internalization of more than 1 × 10^9^ boron atoms per cell, which is sufficient for performing BNCT. Boron-containing conjugates of GL44 and Waz 2′-F-RNA efficiently inhibited the growth of U-87 MG human glioblastoma cells after irradiation in model BNCT experiments.

## 2. Results

### 2.1. 2′-F-RNA Aptamers Capable of Internalizing into U-87 MG Human Glioblastoma Cells

In this study, we employed two different 2′-F-RNA aptamers for targeted delivery of boron clusters into U-87 MG human glioblastoma cells. As we showed in our previous work, a 2′-F-RNA aptamer GL44 internalizes specifically into U-87 MG cells, either alone or as a covalent 5′-conjugate with *closo*-dodecaborate [11].

**Figure 1:**
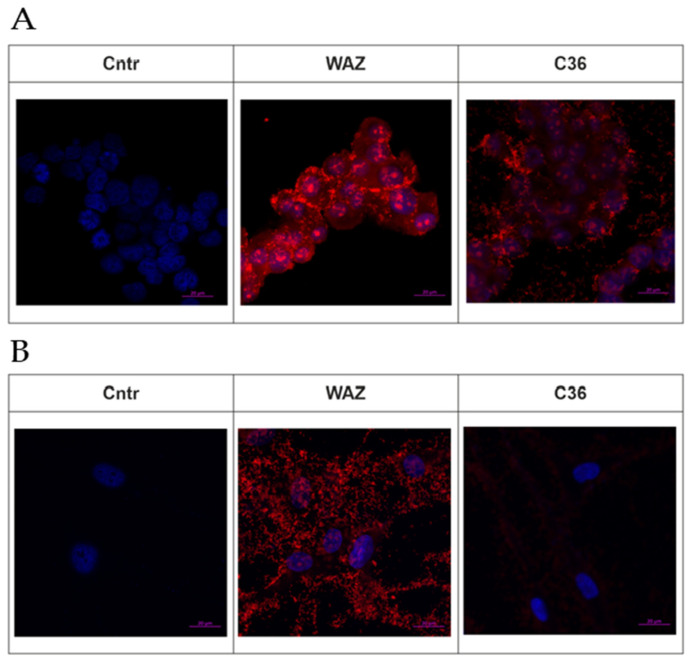
Cytochemical staining of U-87 MG human glioblastoma cells (**A**) and normal human fibroblasts hFF8 (**B**) using a SulfoCy5-labeled Waz 2′-F-RNA aptamer and scrambled control RNA C36 (see Materials and Methods section (Section 4.5) for abbreviations and nucleotide sequences). DAPI-stained nuclei, blue signal; Cy5-labeled aptamers and scrambled controls, red signal.

In the present research, we also used a Waz aptamer that is specific to the transferrin receptor (TfR) on a cell surface [20]. Bearing in mind future in vivo studies, we chose this aptamer for its potential capability of TfR-mediated penetration through the blood–brain barrier. We supposed that a Waz aptamer could act as a component of multi-functional constructs that provide a systemic delivery. It has also been reported that the Waz aptamer can internalize into the cells expressing hTfR on the surface and can also deliver cargo molecules [21]. In particular, TfR is overexpressed in malignant brain tumor cells, and addressing TfR has been employed in the development of targeted therapeutics [22]. Therefore, we decided to investigate the interaction of a Waz aptamer with the U-87 MG cells used in this study. Confocal fluorescent microscopy of U-87 MG cells treated by a Sulfo-Cy5 conjugate of Waz showed that the aptamer efficiently internalizes into the target cells. The conjugate was visualized in cytoplasm as well as inside the nuclei and nucleoli (Figure 1A).

On the contrary, control scrambled 2′-F-RNA oligonucleotide C36 (sequence taken from [20]) demonstrated weak penetration into the cytoplasm and nuclei of tumor cells. Non-specific adherence of control 2′-F-RNA on the plate wells may suggest the non-specific cell binding.

Meanwhile, the confocal microscopy of normal hFF8 fibroblasts treated with the same aptamer showed that Waz very poorly penetrated into the cytoplasm and nucleoli, as compared to U-87 MG cells (Figure 1B). An analysis of the Z-stack indicates the nonspecific adhesion of the aptamer to the surface of the cell and glass slide. The control scrambled oligonucleotide C36 2′-F-RNA almost did not penetrate into the hFF8 cells.

Taking these results together, we consider Waz as a promising candidate for boron cluster delivery into tumor cells.

### 2.2. Synthesis of Conjugates of 2′-F-RNA Aptamers with Closo-Dodecaborate

We showed in the previous study that attaching a boron cluster to the 5′-end of a GL44 aptamer and treating glioblastoma cells with this conjugate with subsequent irradiation by an epithermal neutron beam leads to a specific decrease in cell viability [11]. For the further development of this research, we needed to know if the particular site of boron cluster localization (5′- or 3′-terminal) affects the aptamer-driven cell delivery. We also questioned whether there were any preferences in terms of cluster localization for different aptamers with various nucleotide sequences and spatial structures. Therefore, we studied two different 2′-F-RNA aptamers, GL44 and Waz, that were capable of specifically internalizing into U-87 MG cells. *Closo*-dodecaborate residues were attached either to the 3′- or to the 5′-terminus of each aptamer. The same conjugates were obtained for scrambled control 2′-F-RNA (Fscr-3B12 and Fscr-5B12).

Terminal 5′- and 3′-conjugates with *closo*-dodecaborate were synthesized via the method we proposed earlier in [23], through the reaction of the azido derivative of *closo*-dodecaborate with 5′- and 3′-alkyne modified 2′-F-RNA oligonucleotides. The nucleotide sequences and structures of the conjugates and their characteristics are provided in the Materials and Methods section (Section 4.5).

### 2.3. Evaluation of Cell Penetrating Efficiency for Boron-Containing Aptamer Conjugates

Sufficient boron-10 levels inside target cells is one of the key factors of efficient BNCT. As a rule, it is evaluated as 20 µg/g or 10^9 10^B atoms per cell [6,7]. We have demonstrated that the 2′-F-RNA aptamers used in this study specifically accumulate in U-87 MG glioblastoma cells. Meanwhile, confocal microscopy employed in those experiments did not allow for the quantitative assessment of boron concentration. Therefore, it was of interest to evaluate the amount of boron atoms that were delivered as a cargo of cell-specific aptamers. For this purpose, we used two independent methods: (1) evaluating the amount of 2′-F-RNA carrying the boron cluster and (2) determining the amount of boron atoms. In both cases, we treated the cells with the conjugate GL44-5B12, washed out the unbound molecules and examined the amount of internalized molecules. Details of both assays are reported below.

#### 2.3.1. Evaluation of Intracellular Level of 2′-F-RNA Aptamer–Boron Cluster Conjugate by qRT-PCR

In our case, the aptamer is represented by 2′-F-RNA, which allows the possibility to detect it via qRT-PCR with specific primers, by comparison with [24]. We took for the assay a 2′-F-RNA aptamer with a 5′-modification to leave its 3′-end free for the hybridization with a reverse primer. The average value calculated from the RT-PCR data was found to be (3.51 ± 0.25) × 10^8^ aptamers/cell. Each aptamer bears a cluster made of 12 boron atoms, so the total amount of delivered boron can be estimated as 4.2 × 10^9^ atoms, which is sufficient for BNCT. However, qRT-PCR-based measuring is an indirect approach, whose protocol includes several steps with intermediate RNA isolation and precipitation and renders a rather rough estimation. A more accurate evaluation of aptamer-delivered boron amount can be performed by directly measuring boron atoms by atomic emission spectroscopy.

#### 2.3.2. Evaluation of Intracellular Level of 2′-F-RNA Aptamer–Boron Cluster Conjugate by Direct Boron Measurement

The same aptamer conjugate, GL44-5B12, was used for direct boron determination. For highly sensitive boron measuring, we employed inductively coupled plasma–atomic emission spectrometry (ICP-AES). It is important to emphasize that we performed nearly all the steps of sample processing before analysis in a one-pot manner, without extractions and precipitations, thus minimizing the loss of the conjugate during the intermediate steps. An average boron content normalized on the cell number was found to be (1.5 ± 0.03) × 10^9^ boron atoms per cell. This result nicely coincided with the value obtained from qRT-PCR assay. Since ICP-AES assay is direct and nearly free of any loss of the analyzed compound, we consider the obtained value as more accurate.

Therefore, an assessment of aptamer-delivered boron inside target cells made by two independent methods showed a boron level compatible with generally accepted criteria for BNCT efficiency.

### 2.4. The Effect of Aptamer–Boron Cluster Conjugate on Cell Viability after Irradiation

In the next step of the study, we performed model BNCT experiments using 2′-F-RNA aptamers GL44 and Waz with boron clusters at the 3′ or 5′-end. We deliberately used *closo*-dodecaborate residues made of natural boron here, which comprise 20% of ^10^B isotope. The isolation of pure ^10^B isotope is quite an expensive process that requires special manufacturing. The use of natural boron (a 1:4 mixture of stable ^10^B and ^11^B isotopes) may significantly simplify the production of boron compounds for BNCT and make it much more cost-effective. Therefore, we examined aptamers loaded with natural boron clusters as BNCT agents in this study.

As a positive control for BNCT, we used ^10^B-boronophenylalanine. The cell specificity of aptamer-based boron delivery was evaluated using: (1) human fibroblasts as control non-malignant cells and (2) boron-loaded 3′ and 5′-conjugates of scrambled 2′-F-RNA (Fscr-3B12 and Fscr-5B12) as non-targeted boron delivery agents. Cell viability after the irradiation was examined by two methods: real-time monitoring of cell proliferation and clonogenic test (see next subsections).

#### 2.4.1. The xCELLigence Real-Time Cell Analysis (RTCA)

**Figure 2:**
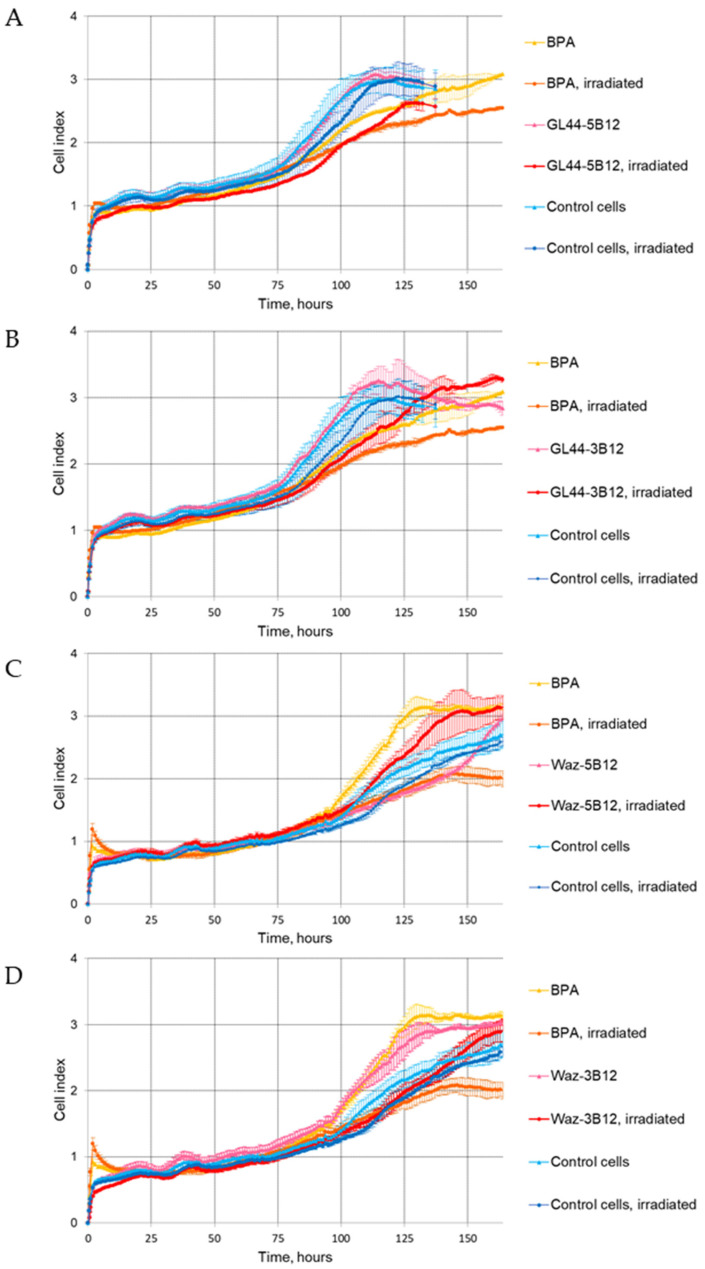
Dynamic RTCA monitoring of cell adhesion and proliferation of U-87 MG human glioblastoma cells after pre-treatment with boron-containing 2′-F-RNA conjugates GL44-5B12 (**A**), GL44-3-B12 (**B**), Waz-5B12 (**C**) or Waz-3B12 (**D**), with or without subsequent irradiation with epithermal neutrons. Irradiated cells pre-treated with ^10^B-BPA were used as a positive control. Control cells—U-87 MG cells that were not incubated with any boron compounds. Parametric data are expressed as mean ± standard deviation (SD). Each experiment was repeated at least two times due to the limitation of the number of wells in the E-Plate.

The xCELLigence RTCA system was used to assess cell proliferation in real time after incubation with boron-loaded aptamers and subsequent irradiation with epithermal neutrons. In the case of the GL44 aptamer, both conjugates with *closo*-dodecaborate, GL44-5B12, and GL44-3B12, caused a statistically significant decrease in the cell index (CI) for U-87 MG, as compared to aptamer-treated cells without irradiation. The effect lasted for up to 120 h of monitoring (Figure 2A,B) and evidences the selective inhibition of cell viability caused by the simultaneous use of a boron-loaded aptamer and neutron irradiation.

On the contrary, when a boron cluster was attached to the Waz aptamer, the effect of neutron irradiation depended on the particular site of attachment. The 3′-conjugate with *closo*-dodecaborate decreased CI values, as compared to non-irradiated U-87 MG cells, over 140 h of monitoring (Figure 2D). However, for a boron cluster attached to the 5′-terminus of Waz, we did not register lower U-87 MG cell viability after irradiation. In this set of experiments, the CI curve for Waz-5B12-treated, but non-irradiated, cells showed even lower CI values as compared to analogous irradiated samples (Figure 2C). We attributed this inconsistency to the possible minor inaccuracies during cell transfer from the vial to the xCelligence E-plate. Similar runs of CI curves for Waz-5B12-treated and irradiated cells and for control cells without aptamers also point to the absence of the cell-inhibiting effects of Waz-5B12. For proving this observation, we employed an independent assessment of cell viability via a clonogenic assay (see Section 2.4.2 below).

It is also worth noting that proliferation curves for U-87 MG cells irradiated in the presence of BPA were similar to the CI curves for the boron-loaded aptamers GL44-5B12 and GL44-3B12 over 100 h of monitoring (Figure 2A,B) and for Waz-3B12 over 140 h of monitoring (Figure 2D). However, at the end of the observation, the CI values of cells irradiated in the presence of BPA were the lowest, indicating a longer inhibitory effect.

**Figure 3:**
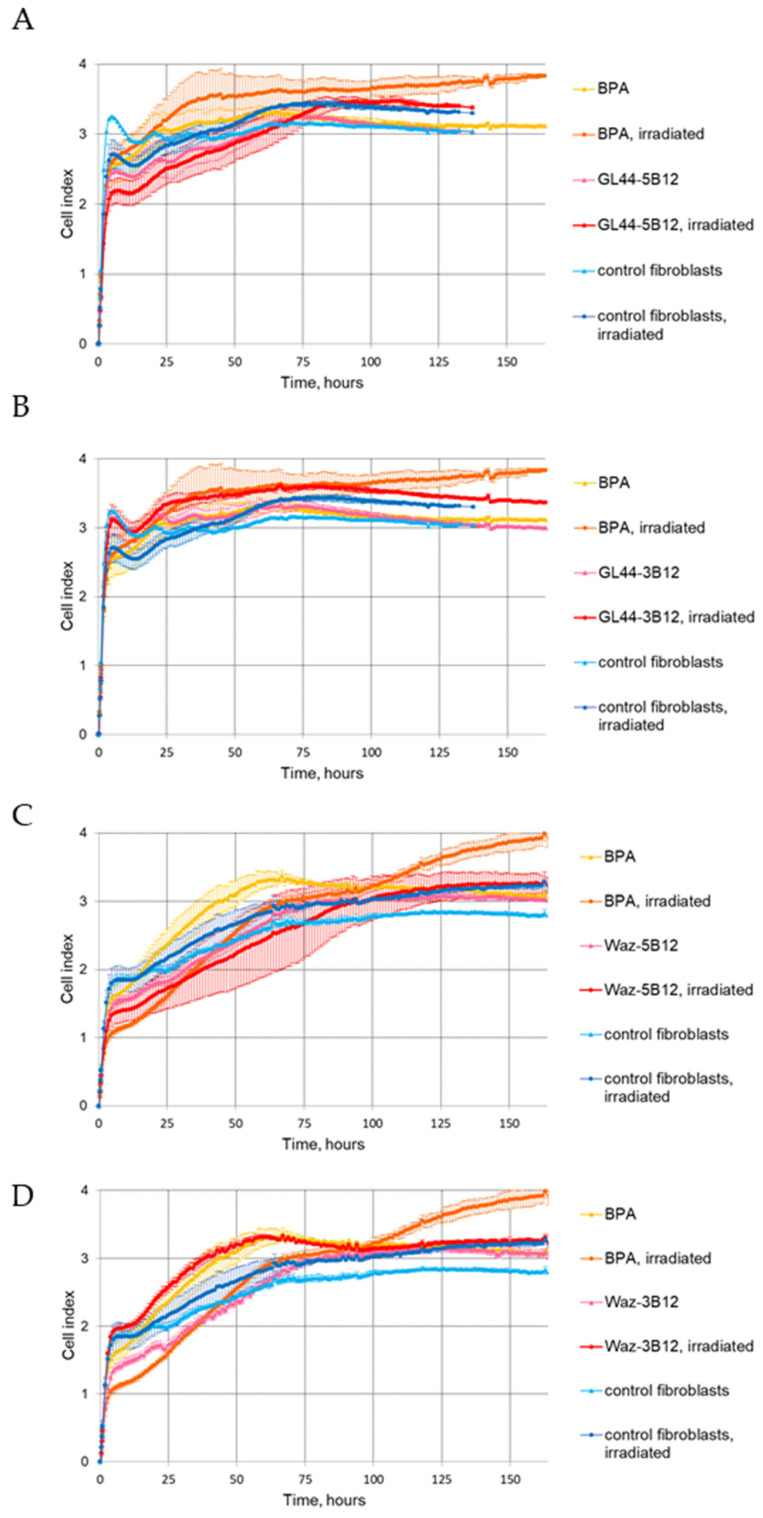
Dynamic RTCA monitoring of cell adhesion and proliferation of hFF8 human fibroblasts after pre-treatment with boron-containing 2′-F-RNA conjugates GL44-5B12 (**A**), GL44-3B12 (**B**), Waz-5B12 (**C**) or Waz-3B12 (**D**), with or without subsequent irradiation with epithermal neutrons. Irradiated cells pre-treated with ^10^B-BPA were used as a positive control. Control cells—human fibroblasts that were not incubated with any boron compounds. Parametric data are expressed as mean ± standard deviation (SD). Each experiment was repeated at least two times due to the limitation in the number of wells in the E-Plate.

To prove that the observed effects are cell-specific, we also performed BNCT experiments on non-cancerous cells, human fibroblasts hFF8, and evaluated their proliferation via real-time cell monitoring as described above. The cells irradiated after treatment with the aptamers GL44-5B12 (Figure 3A), GL44-3B12 (Figure 3B), Waz-5B12 (Figure 3C) and Waz-3B12 (Figure 3D) showed CI curves similar to those for aptamer-free or non-irradiated cells.

#### 2.4.2. Clonogenic Assay

We carried out an independent assessment of the survival of U-87 MG cells treated with aptamers and after irradiation, using the classical clonogenic assay to test the ability of cells to form colonies. This method is most often used for in vitro BNCT model studies. Control non-tumor fibroblast cells are unable to form clearly differentiated colonies, and, for them, the assay was not applicable.

**Figure 4:**
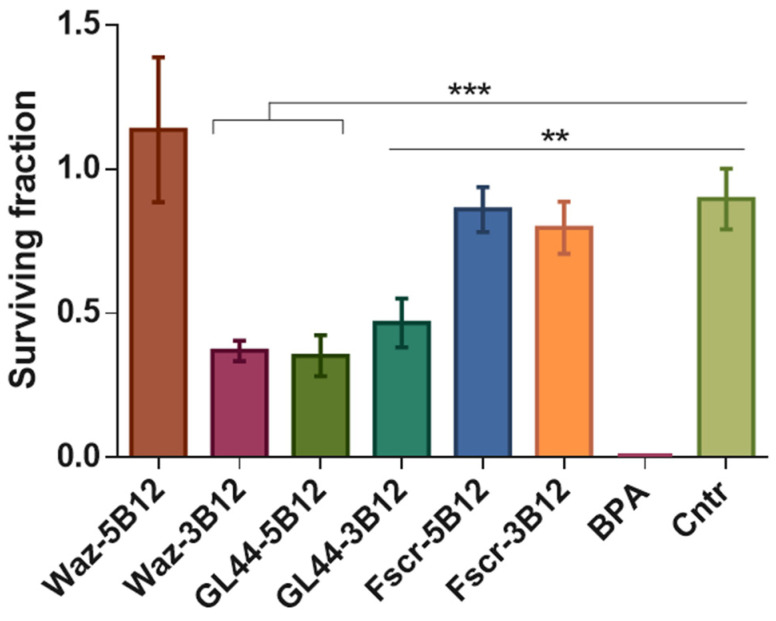
Surviving fractions of neutron-irradiated U-87 MG human glioblastoma cells depending on the incubation with the boron-containing 2′-F-RNA aptamer conjugates Waz-5B12, Waz-3B12, GL44-5B12 or GL44-3B12 and the scrambled 2′-F-RNA conjugates Fscr-5B12, Fscr-3B12 or BPA. Control cells (Cntr) were irradiated without pre-treatment with any boron compounds. ** *p* ≤ 0.01; *** *p* ≤ 0.001. Three replicates were carried out in each case.

The assay has been analyzed by commonly used plating efficiency-based normalization (Figure 4). After irradiation with epithermal neutrons, the surviving fraction of U-87 MG cells treated with BPA was close to zero. Both *closo*-dodecaborate conjugates of GL44 aptamer, GL44-5B12, and GL44-3B12, caused prominent, statistically significant decreases in cell viability, as well as the 3′-conjugate of the Waz aptamer (Waz-3B12), although their effect was weaker than that of BPA. Both scrambled 2′-F-RNA controls with a boron cluster attached either to the 5′ (Fscr-5B12) or 3′-end (Fscr-3B12) showed the same viability as control boron-free cells, which further proves the specificity of boron-loaded aptamers. Notably, the Waz aptamer bearing a boron cluster at the 5′-end showed no difference from the control boron-free cells and scrambled 2′-F-RNA controls. The data obtained provide reliable proof for the results obtained by RTCA analysis and show that physiologically relevant concentrations of boron cluster-conjugated aptamers reduce cell viability in BNCT model experiments. Otherwise, the location of the terminal boron cluster does influence the effect of BNCT. In particular, an improper cluster location for a certain aptamer can totally diminish its functional activity.

## 3. Discussion

Despite significant progress in the development of targeted boron delivery agents of BNCT, the search for novel compounds that are precisely addressed, non-toxic and non-immunogenic and loaded with a sufficient amount of boron still remains an acute task. In our previous research, we demonstrated the possibility of employing a 2′-F-RNA aptamer specific to human glioblastoma cells for the targeted delivery of boron clusters and model BNCT [11]. In that study, *closo*-dodecaborate residue was attached to the 5′-terminus of the aptamer. However, our experiments with model oligonucleotides bearing terminal boron clusters showed that, in some cases, the cluster itself can influence the structure of complementary complexes of such modified nucleic acids [23]. Therefore, we found it reasonable to examine whether the functional activity of a boron-loaded addressing aptamer depends on the particular localization of the cluster.

We have chosen two aptamers for targeted internalization into U-87 human glioblastoma cells. The 2′-F-RNA aptamer GL44 was employed in our previous study on model aptamer-addressed BNCT and demonstrated the ability to enter target cells, with further accumulation in nuclei and nucleoli [11]. In the present study, we also employed a 2′-F-RNA Waz aptamer specific to the transferrin receptor [12] for two reasons. First, the transferrin receptor is expressed on the surface of glioblastoma cells and, in particular, U-87 MG cells. Moreover, Waz binding with the receptor is followed by its cell internalization [20]. Second, the molecules that specifically bind TfR represent potential brain delivery agents that cross the blood–brain barrier [25,26,27], which is especially important for the development of glioblastoma-targeting agents. We supposed that a Waz aptamer could possibly bring a double advantage, both for brain delivery and for targeted transport into glioblastoma cells. Our confocal microscopy experiments with fluorescently labeled Waz showed its specific and effective internalization to U-87 MG cells with nuclear localization, which encouraged us to conduct further studies.

Using the approaches developed in our previous works [11,23], we synthesized and characterized the conjugates of GL44 and Waz 2′-F-RNA aptamers with *closo*-dodecaborate attached at the 3′or 5′-end. While we have already shown that the 5′-conjugate of GL44 works as a boron delivery agent in the in vitro BNCT, the amount of boron atoms brought into the cell still remained unknown. Here, we quantitatively evaluated the aptamer-delivered boron amount in U-87 MG cells via an example with the same conjugate. According to the chemical nature of the conjugate, we used two independent methods of assessment: qRT-PCR for the 2′-F-RNA part and ICP-AES for the boron cluster. The results obtained by both approaches are in good agreement. Atomic emission spectroscopy allows direct measuring of cell-internalized boron; therefore, we consider the value determined by this method to be more accurate. Estimates from both methods render a boron amount per one cell of more than 1·10^9^ atoms, which is close to the generally accepted threshold for sufficient BNCT.

BNCT experiments included cell treatment with two different aptamers with 5′- or 3′-attached boron clusters. Both conjugates of the GL44 aptamer provided a specific decrease in cell viability after neutron irradiation. In contrast, in the case of the Waz aptamer conjugates, only the 3′-conjugate with *closo*-dodecaborate decreased cell viability after irradiation. The 5′-conjugate did not affect cell growth, which is most clearly visible in the clonogenic assay results (Figure 4). We hypothesize that *closo*-dodecaborate at a 5′-end of a Waz aptamer may disturb the proper spatial structure of the aptamer necessary for receptor binding and subsequent internalization. Most probably, each aptamer requires individual trial-and-error adjustment of boron cluster localization. Importantly, neither of the boron-loaded aptamers affected the viability of control non-tumor cells (human fibroblasts) after neutron irradiation, which suggests the tumor cell-specific effect of BNCT.

In this study, we evaluated cell viability after irradiation via two independent methods. A clonogenic assay is the method of choice for model BNCT studies on cell cultures. The results of this test provide an integral estimation of the irradiation effect. Otherwise, cell index monitoring in real time (RTCA) with an xCelligence represents a powerful up-to-date method that allows us to follow the dynamics of cell growth and permanence of irradiation effects. However, RTCA is more sensitive to the experimental conditions, and 10–15% variations in cell index are considered as normal for the assay. Small experimental inaccuracies during cell transfer to xCelligence wells may distort the run of the cell index curve, which hampers the comparison with other curves in the series and the adequate estimation of the whole series [28]. Meanwhile, real-time monitoring allows for the assessment of the dynamics of the inhibiting effect and for the proposal of changes to the experimental protocol. In particular, the restoration of cell viability over prolonged monitoring times shows the necessity to further optimize aptamer treatment regimens and the neutron irradiation of target cells. The combination of real-time cell index monitoring and simple and reliable clonogenic assay made it possible to draw conclusions about the specific inhibition of glioma cells’ growth by three of the four 2′-F-RNA aptamer conjugates examined in this study.

Further research in the field will be directed towards optimizing the dosing and irradiation regimens for boron-loaded aptamers and evaluating their effects in the BNCT experiments in vivo with animal models of glioblastoma.

## 4. Materials and Methods

### 4.1. Chemicals and Reagents

The 5′,N-protected 2′-O-TBDMS-ribo- (A and G), Spacer Phosphoramidite 18, 3′-alkyne-Modifier Serinol polymer support and 3′-PT-Amino-Modifier C6 polymer support were purchased from Glen Research Inc (Sterling, VA, USA). The 5′,N-protected 2′-deoxy-2′-fluoro pyrimidine phosphoramidites and CPG supports were purchased from ChemGene Corp (Wilmington, MA, USA). The N,N-diisopropylethylamine (DIPEA) and propargylamine were purchased from Sigma-Aldrich (St. Louis, MO, USA). The N,N′-disuccinimidyl carbonate (DSC) was purchased from Acros Organics (Geel, Belgium). The Sulfo-Cyanine 5 NHS ester, 10 mM Cu(II)-TBTA stock in 55% DMSO and ascorbic acid were purchased from Lumiprobe (Moscow, Russia), and the sodium dodecaborate Na_2_[B_12_H_12_] was from AviaBor (Dzerzhinsk, Russia). The ^10^B-enriched (>99%, BPA) was purchased from Katchem spol. s r. o. (Prague, Czech Republic) and converted into fructose 1:1 complex for increased solubility [29]. All solvents (THF, DMSO, CH_3_CN (various vendors)) were dried using 3 Å molecular sieves or by distillation and stored over CaH_2_. Bis-tetrabutylammonium-(4-azidobuthoxy)-undecahydro-*closo*-dodecaborate (*closo*B12-azide) was synthesized as described in [23] and kindly provided by Dr. V.N. Silnikov (ICBFM SB RAS).

### 4.2. Cell Lines

The cancer cell line U-87 MG was received from the Russian cell culture collection (Russian Branch of the ETCS, St. Petersburg, Russia). Control non-cancerous cells, normal human fibroblasts hFF8, were kindly provided by Dr. M.L. Filipenko (ICBFM SB RAS, Novosibirsk, Russia). U-87 MG and hFF8 cells were cultivated in Minimum Essential Media (MEM) and Iscove’s Modified Dulbecco’s Medium (IMDM) (Gibco, Waltham, MA, USA), respectively, supplemented with 10% of fetal bovine serum (Gibco, Waltham, MA, USA), 1 mM L-glutamine and 1% (*v*/*v*) antibiotic–antimycotic solution (Gibco, Waltham, MA, USA). Cells were grown in a humidified 5% CO_2_–air atmosphere at 37 °C and were passaged with TripLE Express Enzyme (Thermo Fisher Scientific, Waltham, MA, USA) every 3–4 days.

### 4.3. Synthesis of 2′-F-RNA Aptamers

The 2′-F-RNA aptamers, control scrambled oligonucleotides (C36, GGCGU^F^AGU^F^GAU^F^U^F^AU^F^GAAU^F^C^F^GU^F^GU^F^GC^F^U^F^AAU^F^AC^F^AC^F^GC^F^C^F^; fscr, AC^F^U^F^GGU^F^AU^F^GU^F^C^F^GAGC^F^C^F^AAC^F^AAU^F^C^F^GAU^F^AC^F^C^F^AAGAC^F^U^F^AAGA) and DNA primers were synthesized by the solid phase phosphoramidite method at the 0.4 µmol scale on an automated DNA/RNA synthesizer ASM-800 (Biosset, Novosibirsk, Russia) using corresponding 5′,N-protected phosphoramidites of deoxynucleosides, 2′-O-tert-butyldimethylsilyl (2′-O-TBDMS) ribonucleotides and 2′-fluoro-2′-deoxyribonucleotides, by the protocols optimized for the instrument. Oligonucleotides bearing a 3′-amine group were synthesized using modified polymer support 3′-PT-Amino-Modifier C6 CPG and Spacer Phosphoramidite 18. Oligoribonucleotides bearing a 3′-alkyne group were synthesized using a modified polymer support 3′-alkyne-Modifier Serinol CPG (Glen Research, Sterling, VA, USA). Oligonucleotides with 5′-alkyne modification were obtained in analogy with [30]. After synthesis, the polymer-bound protected oligodeoxyribonucleotides were treated for 15 min with 300 μL of 40% aq. methylamine solution at 65 °C for cleavage from support and deprotection of heterocyclic bases and phosphate groups. The deprotection of 2′-F-RNA oligonucleotides was carried out with 300 μL of AMA solution (NH_4_OH/40% aq. CH_3_NH_2_ 1:1 *v*/*v*) at 25 °C for 2 h. To remove the 2′-O-protective groups (2′-O-TBDMS) from ribonucleotides, we used 200 μL of mixture NMP/TEA·3HF/TEA (150/100/75) at 65 °C for 1.5 h, then treated the solution with 300 μL of trimethylethoxysilane (TCI, Portland, OR, USA) and precipitated oligoribonucleotides by diethyl ether.

### 4.4. Synthesis of Sulfo-Cy5-Labeled 2′-F-RNA Aptamers

To attach the fluorescent label to the 3′-terminus of the 2′-F-RNA Waz aptamer or scrambled control 2′-F-RNA C36, a solution of 1 mg (1.5 µmol) of N-hydroxysuccinimide ester of Sulfo-Cyanine 5 in 80 μL of DMSO was added to the 20 μL of the solution of 3′-amino modified 2′-F-RNA (25 nmol) in a 50 mM Tris-HCl buffer (pH 7.8). The reaction was carried out for 2 h at room temperature. The resulting conjugates were precipitated as Na^+^ salts by the 2% sodium perchlorate in acetone. The fluorescent conjugate of 2′-F-RNA was isolated by 15% denaturing polyacrylamide gel electrophoresis (PAGE) in the 0.4 mm gel, followed by elution from the gel with 0.3 M NaClO_4_ solution, desalted with a Sep-Pak C18 cartridge (Waters, Milford, MA, USA) and precipitated as sodium salts. The homogeneity of the purified conjugate was proved by the denaturating 15% PAGE.

### 4.5. Synthesis of 2′-F-RNA Conjugates with Closo-Dodecaborate

An azido derivative of *closo*-dodecaborate was attached to oligonucleotides as described in [23], starting from 3′- or 5′-alkyne-modified 2′-F-RNAs. The conjugates were isolated by 15% PAGE in the 0.4 mm gel, followed by elution from the gel with 0.3 M NaOAc solution and precipitation with ethanol as sodium salts. Purified oligonucleotide conjugates were characterized by ESI mass spectrometry (Table 1).

### 4.6. Confocal Microscopy

Fluorescent staining of U-87 MG and hFF8 cells was carried out according to the technique described in [31], with minor modifications. The cells were grown on BD Falcon chambered culture slides to 80–90% confluence. Fluorescently labeled 2′-F-RNAs (200 nM in αMEM) were heated to 95 °C for 5 min, ice-cooled for 2 min and incubated at 37 °C for 15 min. The cells were washed with PBS twice and incubated with 2′-F-RNA aptamer Waz or scrambled control 2′-F-RNA C36 for 30 min at 37 °C in the dark at 50 rpm and then washed five times with 250 μL PBS. After that, 200 μL per well of cold methanol was added and incubated for 10 min at 4 °C. Next, the cells were washed twice with 250 μL cold PBS, incubated with 2.5 μM CellTracker™ green CMFDA (5-chloromethylfluorescein diacetate) dye at 37 °C in the dark and again washed twice with PBS. The cells were stained with DAPI (Thermo Fisher Scientific, USA) and visualized by fluorescent microscopy Axioskop 2 Plus (Zeiss, Jena, Germany) at the Center for Microscopic Analysis of Biological Objects of SB RAS (Novosibirsk, Russia). The background level of cell autofluorescence was determined from unstained controls. The operating value of the exposure for the Cy5 channel was the same for all the analyzed samples and amounted to 760 ms.

### 4.7. Evaluation of Cell Internalization of 2′-F-RNA Aptamer

#### 4.7.1. Quantitative RT-PCR Assay

**Figure 5:**
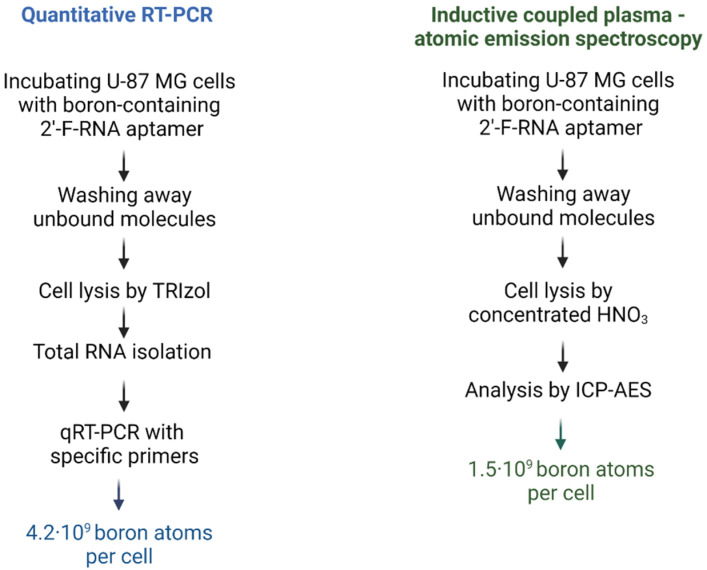
Scheme and results of the qRT-PCR assay and ICP-AES measuring for the internalization of boron-containing aptamer GL44-5B12 into U-87 MG glioblastoma cells.

The course of the experiment is shown in Figure 5 (left panel). U-87 MG cells were grown in 6-well plates to 80–90% confluence and then washed with PBS twice. Each boron-containing 2′-F-RNA conjugate was folded by heating at 95 °C for 5 min and was then incubated at 37 °C for 30 min. Folded 2′-F-RNA aptamers (1 µM in αMEM) were incubated with U-87 MG cells for 45 min at 5 % CO_2_. Unbound and cell-surface-bound 2′-F-RNAs were washed away with PBS supplemented with 0.5 M NaCl. Internalized RNA was isolated along with total RNA using a TRIzol reagent containing 1 pmol per sample of non-related O78t 2′-F-RNA aptamer [32] as a reference control. Quantitative RT-PCR (qRT-PCR) was performed by using the Biomaster RT-PCR-Standard Kit (Cat# RM03-400) from Biolabmix (Novosibirsk, Russia) with Rotor-Gene Q 5plex PCR cycler (Qiagen, Hilden, Germany). All the reactions were performed in 25 µL in triplicate. For each RNA sample, we performed two different RT-PCR reactions: one with aptamer-specific primers (5′-ACGTTACTCTTGCAA and 5′-GAACTATAAGAGGCTA for the GL44 aptamer; 5′-ACTGGTATGTCGAGC and 5′-TCTTAGTCTTGGTATC for the Fscr control) and the other with reference control-specific primers (5′-GGGAGACAAGAATAA and 5′-GAGCAAGTAAACGGCG). The amount of 2′-F-RNA was calculated using the Rotor-Gene Q software package (version 2.2.2). The data obtained were normalized to cell number.

#### 4.7.2. Inductive Coupled Plasma–Atomic Emission Spectrometry (ICP-AES) Assay

The design of the experiment is depicted in Figure 5 (right panel). U-87 MG cells were grown in 6-well plates to 80–90 % confluence and then washed twice with PBS. On the day of the experiment, 1 × 10^6^ cells were transferred into separate tubes. In preliminary experiments, we measured the basic boron level in the same amount of U-87 MG cells (without treatment with any boron compounds) by ICP-AES and found it to be no higher than the baseline within the measurement accuracy. Boron-containing 2′-F-RNA aptamer GL44-5B12 was folded as described above (2.7.1.) The cells were incubated with GL44-5B12 (10 µM in αMEM) for 45 min with light stirring, then centrifuged and washed twice with 500 µL PBS. Unbound and cell-surface bound 2′-F-RNAs were washed away with PBS. The cells were then decomposed by concentrated nitric acid HNO_3_ (extra pure grade). Each sample was supplied by 1 mL of nitric acid and heated to 80 °C for 2 h in an open vessel to obtain a clear solution. The resulting samples were diluted by MilliQ water up to 5 mL, and scandium Sc (2 ppm) was added as an internal standard.

Total boron content was measured by the inductively coupled plasma–atomic emission spectrometry (ICP-AES) using high-resolution iCAP-PRO spectrometer (Duo) (Thermo Fisher Scientific, USA) with Q-tegra Intelligent Scientific Data Solution Software (Thermo Fisher Scientific, USA) in the Core ‘Multi-elemental and Isotope Research’ Facility of the Sobolev Institute of Geology and Mineralogy SB RAS. The analytical signal was registered with an axial view of plasma. Two analytic lines, B 249.678 nm and Sc 255.237 nm, were used. The instrumental conditions were power: 1150 W, argon flow rate: 0.65 L/min, detection time: 20 s and cooling gas flow rate: 12 L/min. Other instrumental conditions were used according to the manufacturer’s recommendations. Boron determinations were carried out from samples processed in duplicate.

### 4.8. Model BNCT Experiments

U-87 MG human glioblastoma cells (0.5 × 10^6^ cells per well) were incubated with boron-containing 2′-F-RNA conjugates (1.7 μM, 800 μL in αMEM) for 30 min then washed with 1 mL of complete culture medium.

U-87 MG cells incubated with ^10^B-4-borono-L-phenylalanine (20 μg of ^10^B per mL) for 18 h at 37 °C under 5% CO_2_ were used as positive controls. Conversely, irradiated cells and non-irradiated cells that were not incubated with any boron compounds served as negative controls.

Control hFF8 human fibroblasts were treated with boron-loaded 2′-F-RNA conjugates or BPA in the same manner as described above for U-87 MG cells.

Prior to irradiation, all samples were transferred into 2 mL vials and placed in an organic glass phantom. The neutron irradiation of cell cultures was performed at the accelerator-based epithermal neutron source VITA [5] for 1 h at 2 MeV proton energy and 1.4 mA proton current. Epithermal neutron fluence was 5 × 10^11^ neutrons/cm^2^.

### 4.9. The xCELLigence Real-Time Cell Analysis (RTCA)

After cell irradiation for RTCA, U-87 MG tumor cells or control hFF8 fibroblasts were seeded in two eight-well E-plates (3 × 10^4^ cells per well). The impedance value of each well was monitored automatically in real-time with the use of an xCELLigence RTCA system (Agilent Technologies, Santa Clara, CA, USA) over 160 h and expressed as a CI (cell index) value [33]. Parametric data are expressed as mean ± standard deviation (SD). Each experiment was repeated at least two times due to the limitation of the number of wells in the E-plate.

### 4.10. Clonogenic Assay

Irradiated U-87 MG tumor cells were seeded in 6-well plates (TPP, Trasadingen, Switzerland) at a density of 300 cells per well. The cells were incubated for a week at 37 °C in a humidified incubator under 5% (*v*/*v*) CO_2_. A clonogenic assay was carried out according to the technique described earlier [34]. The calculation of the percent plating efficiency and surviving fraction was based on the survival of non-irradiated cells. Three replicates were made in each case.

### 4.11. Statistical Analyses

The outcome variables are expressed as means ± standard deviations (SDs). Each experiment was repeated at least three times. The statistical analyses were performed with GraphPad Prism 6.01 (GraphPad Software, San Diego, CA, USA). For comparisons of more than two sets of data, we used two-way ANOVA. Differences were considered as significant if the *p*-value was <0.05.

## 5. Conclusions

In this study, we have broadened the range of novel BNCT agents, tumor cell-specific aptamers conjugated with *closo*-dodecaborate. We employed here two 2′-F-RNA aptamers addressing human glioblastoma cells: GL44 aptamer, specific to U-87 MG cells, and Waz aptamer, specific to the transferrin receptor. Using 2′-F-RNA aptamer GL44 as an example, we have demonstrated that aptamer-driven delivery provides the accumulation of an amount of boron sufficient for BNCT. In vitro experiments on the epithermal neutron irradiation of human glioblastoma cells and normal human fibroblasts have shown that the particular site of attachment of the boron cluster may dramatically influence the efficacy of BNCT. While both 3′- and 5′-conjugates of the GL44 aptamer provided a specific decrease in tumor cell growth after irradiation, only the 3′-conjugate of the Waz aptamer possessed the necessary activity. Therefore, each addressing aptamer needs its own f optimal boron cluster location. It is worth noting that in this study, we used *closo*-dodecaborate made of natural boron with only 20% ^10^B isotope. The replacement of ^10^B-enriched compounds with natural boron ones is very promising in the context of potential therapeutic applications. Although boron-loaded aptamers showed lower efficacy as compared to BPA, at the level of the living organism, they may possess additional benefits due to precise tumor cell targeting, and their efficacy could be further improved by adjusting the dosage and irradiation regimens.

## Figures and Tables

**Table 1:** Conjugates of 2′-F RNA with *closo*-dodecaborate attached at the 3′- or 5′-terminus.

Name	Sequence, 5′-3′	Molecular Mass	
		Calculated	Found *
Waz-5B12	5′-B_12_-L1-GGGUUCUACGAUAAACGGUUAAUGACCAGCUUAUGGCUGGCAGUUCCC	15,232.1	15,279.1 (+2Na^+^)
Waz-3B12	5′-GGGUUCUACGAUAAACGGUUAAUGACCAGCUU-AUGGCUGGCAGUUCCC-L2-B_12_	15,545.3	15,569.8 (+Na^+^)
GL44-5B12	5′-B12-L1-ACGUUACUCUUGCAACACCCAAACUUUAA-UAGCCUCUUAUAGUUC	14,355.6	14,379.7 (+Na^+^)
GL44-3B12	5′-ACGUUACUCUUGCAACACCCAAACUUUAA-UAGCCUCUUAUAGUUC-L2-B_12_	14,668.8	14,714.3 (+2Na^+^)
Fscr-5B12	5′-B12-L1-ACUGGUAUGUCGAGCCAACAAUCGAUACCAAGACUAAGA	12,712.8	12,713.6
Fscr-3B12	5′-ACUGGUAUGUCGAGCCAACAAUCGAUACCAA-GACUAAGA-L2-B_12_	13,025.9	13,048.6 (+Na^+^)
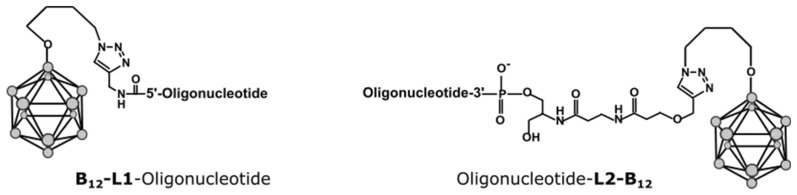			

* m/z values determined by ESI mass-spectrometry. All pyrimidines are 2′-F-modified. The structures of L1 and L2 linkers and *closo*-dodecaborate are shown below in Figure 2.

## Data Availability

Not applicable.

## Abbreviations

AMA	a mixture of ammonium hydroxide and 40% aqueous methylamine
BINP	Budker Institute of Nuclear Physics
BNCT	Boron Neutron Capture Therapy
BPA	Boronophenylalanine
BSH	Sodium borocaptate
CI	Cell Index
DAPI	4′,6-Diamidino-2-phenylindole
DIPEA	N,N-Diisopropylethylamine
DMSO	Dimethyl sulfoxide
DSC	N,N′-Disuccinimidyl carbonate
ESI	Electrospray Ionization
IMDM	Iscove’s Modified Dulbecco’s Medium
ICP-AES	Inductively Coupled Plasma–Atomic Emission Spectrometry
αMEM	Modified Minimal Essential Medium
MTT	3-(4,5-Dimethylthiazol-2-yl)-2,5-diphenyl tetrazolium bromide
NGF	Nerve Growth Factor
TBDMS	Tert-butyldimethylsilyl
TEA	Triethylamine
THF	Tetrahydrofuran
NHS	N-Hydroxysuccinimide
NMP	N-Methyl pyrrolidone
PBS	Phosphate Buffered Saline
RTCA	Real-Time Cell Analysis
TBTA	Tris(benzyltriazolylmethyl)amine
